# Chronic Rheumatism

**Published:** 1854-12

**Authors:** 


Chronic Rheumatism.—Dr Rey has used turpentine vapor baths with great effect in chronic rheumatism, neuralgia, and chronic pulmonary catarrhal affections. The vapor is brought my means of pipes into a room, into which fresh air can be rapidly introduced if necessary. The heat and the amount of turpentine are regulated by circumstances. The flow of sweat is very copious, and the turpentine is also absorbed, and gives the violet odor to the urine. On account of the immense sweating, the baths are very weakening, so that few persons can take more than twelve (on successive days) without leaving them off for some time.—*L'Union.*

